# PET Imaging of the Human Nicotinic Cholinergic Pathway in Atherosclerosis

**DOI:** 10.1007/s11886-015-0614-8

**Published:** 2015-07-17

**Authors:** Matthias Bauwens, Felix M. Mottaghy, Jan Bucerius

**Affiliations:** Department of Nuclear Medicine, Maastricht University Medical Center (MUMC+), Maastricht, The Netherlands; Department of Human Biology, School for Nutrition, Toxicology, and Metabolism (NUTRIM), Maastricht University Medical Center (MUMC+), Maastricht, The Netherlands; Cardiovascular Research Institute Maastricht (CARIM), Maastricht University Medical Center (MUMC+), Maastricht, The Netherlands; Cardiovascular Research Institute Maastricht (CARIM), Maastricht University Medical Center (MUMC+), P. Debyelaan 25, 6229 HX Maastricht, The Netherlands

**Keywords:** Nicotinic acetylcholine receptors, Positron emission tomography, Atherosclerosis

## Abstract

During the past years, non-neuronal vascular nicotinic acetylcholine receptors (nAChRs) increasingly have gained interest in cardiovascular research, as they are known to mediate the deleterious effects of nicotine and nitrosamines, components of tobacco smoke, on the vasculature. Because smoking is a major risk factor for the development of atherosclerosis, it is obvious that understanding the pathophysiologic role of nAChRs in the atherosclerotic disease process, as well as in the development of new diagnostic and therapeutic nAChR-related options, has become more important. Accordingly, we briefly summarize the pathophysiologic role of vascular nAChRs in the atherosclerotic disease process. We also provide an overview of currently available nAChR positron emission tomography (PET) tracers and their performance in the noninvasive imaging of vascular nAChRs, as well as potential nAChR PET tracers that might be an option for vascular nAChR PET imaging in the future.

## Introduction

Interest in non-neuronal vascular nicotinic acetylcholine receptors (nAChRs) has been increasing in cardiovascular research because they are known to mediate the deleterious effects of nicotine and nitrosamines, components of tobacco smoke, on the vasculature. Smoking is a major, if not the greatest, risk factor for the development of atherosclerosis. Therefore, understanding the pathophysiologic role of nAChRs in the atherosclerotic disease process, and consequently, the development of new diagnostic and therapeutic nAChR-related options, has become more important. The first steps in that direction—development of new noninvasive imaging strategies for nAChRs by means of positron emission tomography (PET)—have been taken and, along with the pathophysiologic role of nAChRs in atherosclerosis, are described in this review article.

## Atherosclerosis

The clinical consequences of atherosclerosis—myocardial infarction and stroke—remain the most common causes of death in Europe and worldwide [1–3]. It is well known that atherosclerotic progression and severity are linked to many clinical risk factors, including hyperlipidemia, diet, diabetes, obesity, hypertension, and nicotine abuse.

Atherosclerosis reflects a pathologic process in the arterial wall, where endothelial injury results in accumulation of lipids and inflammatory cells in the intima [1, 4••]. The blood-derived monocytes differentiate into macrophages, which express scavenger receptors, allowing them to ingest oxidized lipids and become foam cells [1, 5]. Early atherosclerotic plaques originate as fatty streaks, which are lipid-filled macrophage foam cells consisting of fat, cholesterol, and other biologic components [4••]. As these lesions further expand, foam cell apoptosis and necrosis result in continuing accumulation of lipids and necrotic debris. This process leads to the development of a highly thrombogenic lipid core and an expansion of the plaque within the arterial wall. In addition, via various signaling cascades, soluble factors are released, mediating platelet adhesion as well as proliferation and migration of vascular smooth muscle cells (VSMCs) from the tunica media to the intima. The VSMCs compose a stabilizing, collagen-rich, fibrous cap, which covers the lipid core, resulting in highly vascularized stable or unstable vulnerable plaques [1, 6].

## Nicotinic Cholinergic Pathway

During recent years, cardiovascular researchers became increasingly interested in nAChRs, the most frequent cholinergic receptors in the central nervous system (CNS), as non-neuronal nAChRs are known to mediate the deleterious effects of nicotine and nitrosamines, components of tobacco smoke, on the vasculature. Because smoking is a major risk factor for the development of atherosclerosis and consequently, stroke and myocardial infarction, it has become clear that understanding the pathophysiologic role of nAChRs in the atherosclerotic disease process, as well as the development of new diagnostic and therapeutic nAChR-related options, is more important than ever. In this review, we focus mainly on the potential of noninvasive imaging of nAChRs by means of PET combined with CT (PET/CT) or MRI (PET/MRI) not on the physiologic or pathophysiologic role of vascular nAChRs in cardiovascular disease; however, we provide a brief summary on the physiology and proatherogenic effects of nAChRs in the vasculature [4••, 7].

nAChRs are a heterogeneous family of ligand-gated transmembrane ion channel receptors that mediate fast synaptic transmission [8–11]. They work as a regulator of excitatory neurotransmission, and nAChR activity in the CNS has been found to regulate physiologic functions such as sleep, fatigue, arousal, central processing of pain, and cognitive functions [8, 10]. From a pathophysiologic point of view, nAChRs were identified as contributing to the pathogenesis of several diseases, including myasthenia gravis, Alzheimer’s disease, and Parkinson’s disease [8, 10, 12]. This discovery led to the development of PET-feasible ^18^F-labeled radiotracers for noninvasive diagnosis of these neurodegenerative disorders [13–15].

### Non-neuronal Vascular nAChRs

It is now known that nAChRs are expressed ubiquitously in almost all cells in the blood vessels. There, these vascular nAChRs accelerate the atherogenic process [4••]. The structure of non-neuronal nAChRs such as vascular nAChRs closely resembles that found in CNS nAChRs: a pentamer composed of distinct subunits arranged tightly around a central pore [7]. So far, 12 different nAChR subunits, α2 to α10 and β2 to β4 have been identified that can form heteromeric nAChRs: for example, α4β2 nAChRs. In contrast, the homomeric receptors (e.g., α7-nAChRs) are made up of five α-subunits.

nAChRs have been found on human VSMCs (α2, α3, α4, α5, α7, α10); human aortic endothelial cells (α3, α5, α7, α10, β2, β3, β4); human platelets (α7); human mononuclear leukocytes (α7, β2); human macrophages (α1, α7, α10); B lymphocytes (α3, α4, α5, α7); and T lymphocytes (α3, α4, α7, β2, β4) [4••, 7, 10, 16–19].

The endogenous ligand for nAChRs is acetylcholine (ACh); however, nicotine and tobacco-specific nitrosamines (e. g., NNK [4-(methylnitrosamino)-1-(3-pyridyl)-1-butanone], NNN [*N*0-nitrosonornicotine], DEN [*N*-diethylnitrosamine]) also are high-affinity nAChR ligands [4••]. Binding of the ligand to the receptor leads to a conformational change in the nAChR, resulting in opening of the ion channel and transmission of the signals. Charged amino acids line the central pore and select the ions that pass through the channel [4••].

### nAChR-Mediated Atherogenic Effects

The non-neuronal nicotinic cholinergic pathway regulates several components of atherogenesis, including inflammation, VSMC phenotype, proliferation, migration, and plaque neovascularization [4••]. The latter process is characterized by a network of capillaries, which originates from the adventitial vasa vasorum and extends into the intimal layer of atherosclerotic lesions [4••].

NNK, a tobacco carcinogen and high-affinity ligand to nAChRs as described earlier, appears to cause endothelial cell apoptosis [4••, 20]. However, these findings are debated, as other groups found NNK to facilitate angiogenesis in lung and gastric cancer [21, 22]. Nicotine promotes chemotaxis and migration of rat and human VSMCs, as well as preventing VSMCs from undergoing apoptosis induced by serum deprivation, suggesting that growth and migration of VSMCs are enhanced by both these pathways [16, 23–27]. Heeschen et al. [28] investigated the effect of nicotine on VSMC migration, proliferation, growth, and neovascularization of advanced atherosclerotic plaques in mice and found a significantly larger median plaque area and a higher percentage of vascularized plaques in nicotine-treated mice compared with a control group. Their results indicated a nicotine-related stimulation of plaque growth related to increasing plaque neovascularization rather than VSMC proliferation [28, 29]. As mentioned earlier, nicotine stimulates VSMC migration depending on nAChR activity [16, 24, 25, 30]. Important signaling pathways that contribute to this nAChR-mediated VSMC migration are elevation of the early growth response gene 1 (Erg-1), increased calcium influx, activation of EGFR, increased telomerase activity, and phosphorylation of Ets-like gene 1 [25, 30, 31]. In addition, nicotine also was found to upregulate levels of proinflammatory cytokines, i.e., IL-6, MCP-1, and NF-κB, in human aortic VSMCs [32].

Nicotine induces the production of growth factors such as TGF-α, TGF-β, PDGF-BB, VEGF, and FGF-2 [25, 33, 34]. These growth factors were shown to stimulate VSMC proliferation (PDGF-BB, TGF-α, and FGF-2) and to promote the migration of human VSMCs and plaque neovascularization (VEGF) [6, 33, 34]. They also cause contractile-to-synthetic transition, cytoskeletal remodeling, enhanced proliferation, and migration of VSMCs—pathways that facilitate formation of the intimal lesions characteristic of atherosclerosis (PDGF-BB) [6, 33, 34]. Furthermore, endothelial permeability appears to be enhanced by increased expression of growth factors. This process also is known to contribute to plaque neovascularization and atherosclerotic progression [4••, 35, 36].

As described earlier, nAChRs are found on several immune cells. The influx of these immune cells, including macrophages, into the arterial wall is known to contribute to atherosclerotic plaque formation [37]. It therefore is likely that nicotine or other stimulating agents act on these immune cells and also on endothelial progenitor cells to facilitate neovascularization of plaques as well as thrombosis [4••]. Nicotine enhances the adhesion of human microvascular endothelial cells to monocytes, a process stimulated by upregulation of the cytokines TNF-α and IL-β [4••, 17]. Furthermore, Aicher et al. [38] showed that nicotine caused a sevenfold increase in proinflammatory cytokine IL-12 and IL-10 levels in human dendritic cells compared with untreated controls [38]. This upregulation was mediated via α7-nAChRs on the dendritic cells. The activation of dendritic cells by nicotine augments their capacity to stimulate T cell proliferation and, as mentioned earlier, to secrete cytokines. Both these pathways may contribute significantly to the proatherogenic effects of nicotine.

## PET Imaging of Vascular nAChRs

PET is the most advanced technique for noninvasive molecular imaging in vivo [39–42]. It allows three-dimensional quantitative detection of the distribution of radiolabeled ligands with high resolution and sensitivity. PET thereby provides functional images of biochemical and physiologic processes in different organ systems [40, 43]. This advanced nuclear medicine technique has been integrated into clinical routine for in vivo imaging mainly in oncology but also in cardiology and neurology and for assessing inflammation [44]. Furthermore, PET is considered a highly sophisticated device for experimental animal research and has been proven to facilitate the translational process in the development of new radiotracers [40, 45, 46]. Clinical PET cameras are now available with a spatial resolution of about 2 to 3 mm, allowing the investigation of radioligand distribution even within small target tissues in both animals and humans [40, 47, 48].

Today, almost all clinical PET systems are equipped with CT within one device, allowing anatomic correlation of the PET findings and therefore the combination of functional (PET) and morphologic (CT) imaging in one session. Whereas “PET-only” systems are now quite rare, the newest development involves hybrid PET/MRI scanners. This challenging imaging technique combining PET and MRI has been used in routine clinical practice for the past few years and has shown much promise in further broadening the spectrum of indication for hybrid functional and morphologic noninvasive imaging.

PET imaging for nAChRs initially was used to evaluate cerebral nAChR distribution in patients with neurodegenerative disorders such as Alzheimer’s and Parkinson’s disease [13–15]. The first attempts focused on imaging cerebral α4β2-nAChRs, which were shown to be significantly reduced in both Alzheimer’s and Parkinson’s disease [15]. Along with further development of ^18^F-labeled PET tracers for clinical imaging of cerebral α4β2-nAChRs, more recent approaches have focused on development and evaluation of α7-nAChR PET tracers, which are now available and awaiting further preclinical and clinical evaluation [49, 50••, 51–53, 54••, 55].

In 2006, our group published the first attempts to image non-neuronal α4β2-nAChRs [56]. It is known that stimulation of the parasympathetic autonomic system decreases heart rate and cardiac contractility and that this pathway is mediated by acetylcholine acting on intracardiac ganglionic nAChRs and cardiac muscarinic acetylcholine receptors (mAChRs). Therefore, we aimed to assess the distribution of cardiac α4β2-nAChRs with a dedicated α4β2-nAChR PET ligand ([^18^F]-2-fluoro-A85380) in humans. Furthermore, we attempted to evaluate whether the cardiac distribution of α4β2-nAChRs differs between healthy controls and patients with known Parkinson’s disease or multiple system atrophy (MSA), as it already was demonstrated that cerebral distribution of these nAChRs is reduced in these patient populations.

Five healthy volunteers without cardiac disease and six patients with either Parkinson’s disease or MSA without additional overt cardiac disease were evaluated with [^18^F]-2-fluoro-A85380 PET imaging to assess cardiac parasympathetic innervation and the putative impact of both disorders thereon. Whole-body PET scans were performed on a Siemens PET/CT Biograph (Siemens, Erlangen, Germany) 75.4 ± 6.7 min after intravenous injection of 371 ± 58 MBq of the tracer. The average count rate density of left ventricular regions of interest (ROIs) and a standard ROI in the right lung was measured in three consecutive slices, and heart-to-lung ratios were calculated consecutively in each volunteer and patient. Heart-to-lung ratios in the volunteer group did not differ from those in patients with Parkinson’s disease or MSA (3.2 ± 0.5 vs. 3.2 ± 0.8 and 2.96 ± 0.7, respectively, mean ± SD; *P* = not significant for all) [56]. Based on the results of our study, we concluded that human cardiac nAChRs can be visualized and measured by [^18^F]-2-fluoro-A85380 PET scans in both cardiac-healthy subjects and patients with Parkinson’s disease or MSA. Furthermore, these first results suggest no significant impact of either Parkinson’s disease or MSA on cardiac nAChRs.

**Fig. 1:**
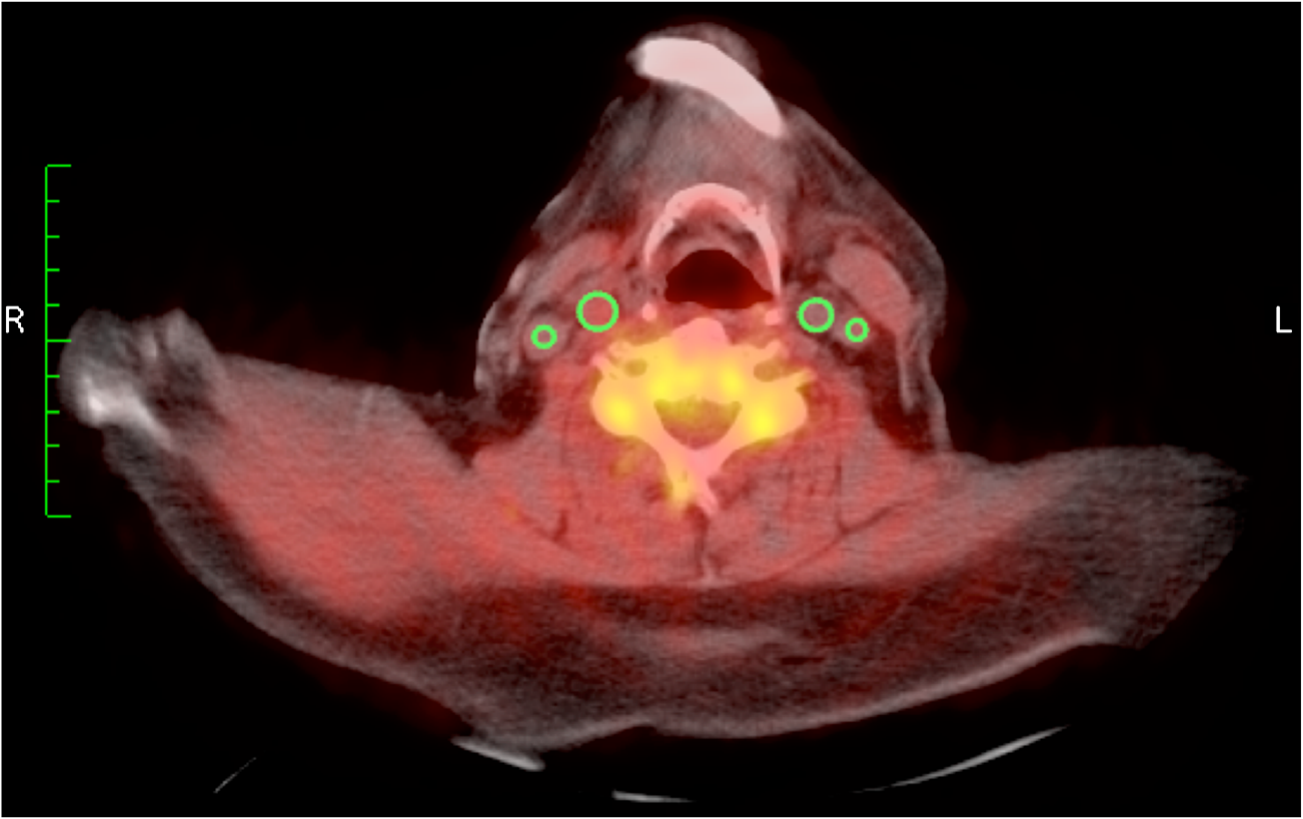
α4β2-nAChR PET/CT scan showing [^18^F]-2-fluoro-A85380 uptake in the right and left common carotid arteries (upper ROIs *green*) and in the right and left jugular veins (lower ROIs). On a visual basis, the fused PET/CT image shows higher uptake of the tracer in the two common carotids compared with the two veins, indicating a specific tracer uptake in the arteries. Furthermore, the right common carotid artery shows a higher uptake (SUV_max_ 1.7 vs. SUV_max_ 1.2 in the left common carotid artery) compared with the left common carotid artery, whereas the uptake in the two jugular veins is almost identical (right SUV_mean_ 1.0 vs. left SUV_mean_ 1.1), further indicating a specific arterial uptake of the PET tracer as well as a higher density of the α4β2-nAChRs in the right than in the left common carotid artery

In a second step, we evaluated the feasibility of the [^18^F]-2-fluoro-A85380 PET tracer for imaging non-neuronal vascular α4β2-nAChRs in the same study population of five healthy controls and six patients with Parkinson’s disease or MSA [57••]. Again, the aim was to determine whether this tracer could noninvasively image vascular α4β2-nAChRs in vivo in humans via PET and to investigate whether neurodegenerative disorders such as Parkinson’s disease and MSA might have an impact on the vascular distribution of these nAChRs. Because of the pathophysiologic importance of nAChRs in atherosclerosis, which was mentioned earlier, proving the ability to noninvasively detect nAChRs in humans is of crucial relevance for the diagnostic and subsequent therapeutic approach in patients with atherosclerosis. We therefore quantified [^18^F]-2-fluoro-A85380 uptake in the ascending and descending aorta, the aortic arch, and the carotids in our aforementioned study population as the maximum target-to-background ratio (Fig. 1). The maximal standardized uptake value (SUV), single hottest segment, and percentage of active segments of [^18^F]-2-fluoro-A85380 uptake in the arteries also were assessed. We could clearly visualize the [^18^F]-2-fluoro-A85380 uptake, and the maximum target-to-background ratio uptake values corrected for the background activity of the tracer showed specific tracer uptake in the arterial walls. Significantly greater uptake values were found in the descending aorta. Comparison between volunteers and patients revealed significant differences, with lower [^18^F]-2-fluoro-A85380 uptake in the patient group when single arterial territories were compared but not when all arterial territories were pooled together [57••]. Our results clearly suggest that [^18^F]-2-fluoro-A85380 can provide specific information on the nAChR distribution in human arteries. Furthermore, the vascular nAChR density appears lower in patients with Parkinson’s disease or MSA, indicating an impact of both neurodegenerative disorders on human arteries [57]. Currently, our group is carrying out studies in larger populations as well as in the experimental setting to further validate the approach of noninvasive in vivo imaging of vascular nAChRs, which will provide more detailed insights into the pathogenic role of nAChRs in the human vasculature [58].

**Fig. 2:**
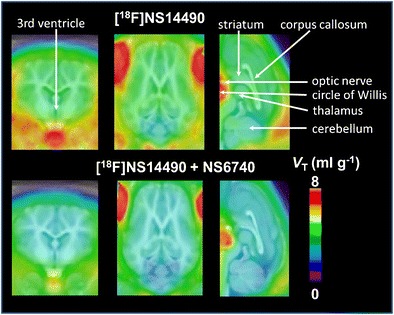
Parametric maps of the distribution volume (*V*_T_) of [^18^F]NS14490 in the brain of piglets. Animal studies were performed without blocked conditions (controls, *upper row*) and with pretreatment with the competitor NS6740 (blocked conditions, *lower row*). Parametric maps are projected onto a magnetic resonance atlas for the pig brain. Values represent the mean of control animals and animals with blocking. Based on the assumption of a complete blockade, the displacement of the α7-nAChR PET tracer by the competitor indicates a specific binding potential in the brain of living pigs, as well as evidence for specific binding in major brain arteries, such as the carotid arteries. The latter appears to enable the PET radiotracer [^18^F]NS14490 to specifically image α7-nAChRs in vulnerable plaques of diseased arterial vessels. Data are given in milliliters per gram [54••]. (With permission from: Rötering S, Deuther-Conrad W, Cumming P, et al.: Imaging of α7 nicotinic acetylcholine receptors in brain and cerebral vasculature of juvenile pigs with [18F] NS14490. EJNMMI Research 2014, 4:43) [89]

Recently, Rötering et al. [54••] evaluated a newly developed α7-nAChR PET tracer ([^18^F]NS14490) preclinically in an experimental pig model. α7-nAChRs are an important molecular target in neuropsychiatry and oncology, but, as mentioned earlier, also play a crucial role in the pathophysiologic disease process in atherosclerosis. So far, the development of applicable highly specific radiotracers has been challenging because of comparatively low protein expression. One novel ligand that might be a feasible candidate for in vivo PET imaging of cerebral but also vascular α7-nAChRs is [^18^F]NS14490. This tracer was shown to yield reliable results in organ distribution studies and therefore was tested in a dynamic PET study in piglets. In this study by Rötering et al. [54••], PET measurements were performed in young pigs to investigate the metabolic stability and cerebral binding of [^18^F]NS14490 without and with administration of the α7-nAChR partial agonist NS6740 in baseline and blocking conditions. Dynamic time–activity curves were calculated for the frontal cortex, temporal lobe, parietal lobe, occipital lobe, hippocampus, striatum, cerebellum, thalamus, middle cortex, ventral cortex, midbrain, pons, colliculi, left carotid artery, and circle of Willis. In addition, to investigate the selectivity of α7-nAChR blockade in the cerebral vessels, SUV in the left carotid artery, and circle of Willis was calculated retrospectively (Fig. 2). Assuming complete blockade of the receptor by the partial agonist, the displacement indicated a specific binding potential of [^18^F]NS14490 in the brain of living pigs, as well as evidence of specific binding in major brain arteries, such as the carotid arteries. The latter is tremendous because it appears to enable the PET radiotracer [^18^F]NS14490 to image α7-nAChRs in vulnerable plaques of diseased vessels [54••]. Within the next few months, a prospective study using the α7-nAChR PET tracer and the recently introduced hybrid PET/MRI technique will begin in patients with carotid artery disease requiring carotid surgery, further evaluating the potential of this PET tracer to image vascular α7-nAChR in atherosclerosis noninvasively.

## Current PET Tracers for α4β2 nAChRs

The concept of imaging the α4β2 receptor, as described earlier, originally was designated for brain receptors. Consequently, several radioligands have been developed, which are discussed in the following text. Not all the tracers listed show high affinity, and even in those that do, it remains to be seen whether they are suitable for imaging vascular nAChRs, as brain studies generally require a different approach. For example, the lipophilicity of a radiopharmaceutical should be high enough to allow it to penetrate the blood–brain barrier (BBB) for imaging of brain receptors. However, this is not a requirement in peripheral imaging; in fact, the lipophilic character of a drug may even lead to slower blood clearance, preventing timely image acquisition protocols. On the other hand, a certain degree of metabolite formation is acceptable in brain receptor imaging, provided the radiometabolites themselves do not cross the BBB. In the periphery, of course, metabolization of the radiopharmaceutical leads to poor image quality and interpretation.
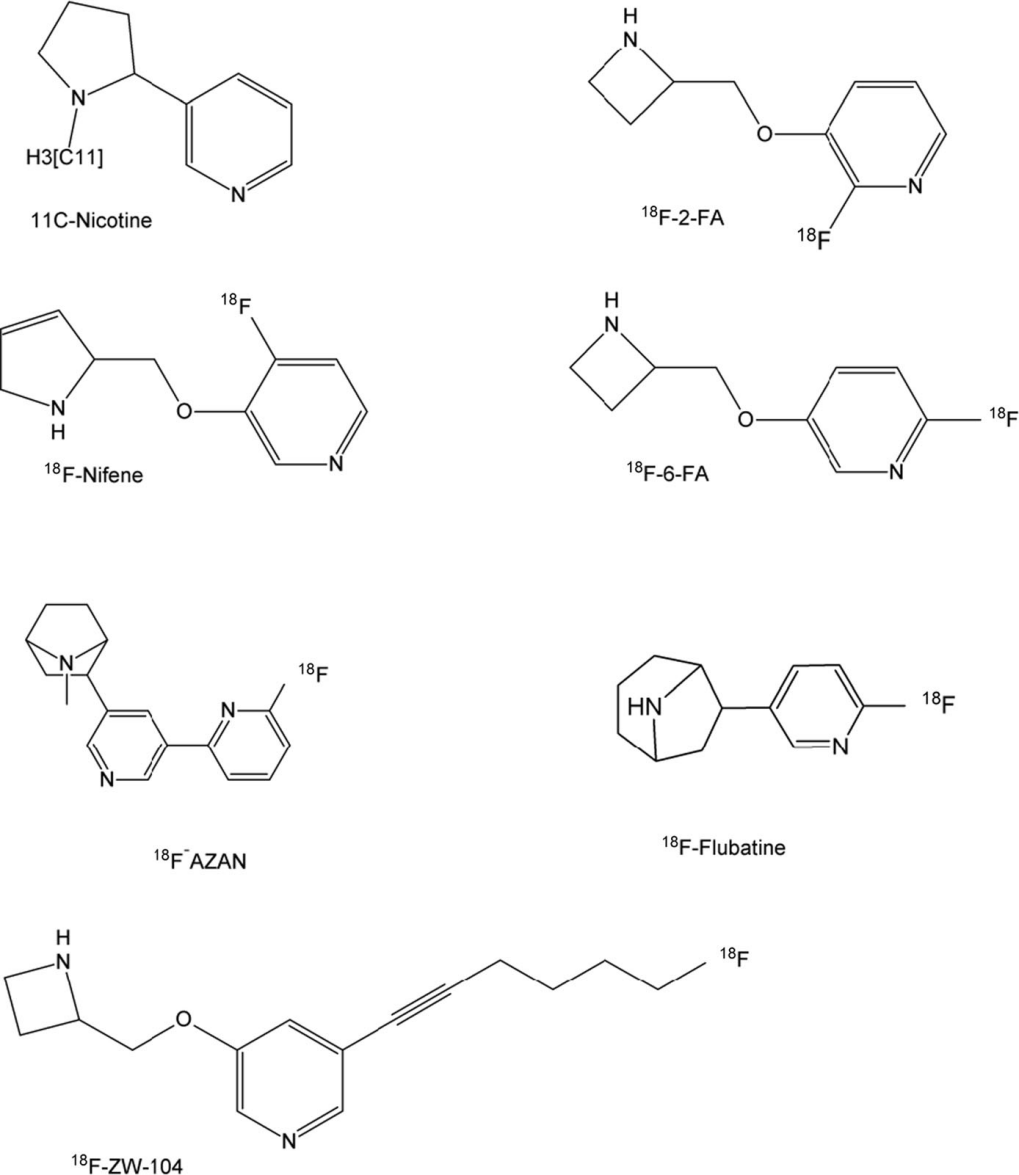


### [^11^C]-Nicotine

[^11^C]-Nicotine was one of the first radioligands used to image cerebral nicotine receptors [59–62]. The radiosynthesis is well-developed, with the latest publication reporting a 90 % decay-corrected yield, 99 % radiochemical purity, and 7- to 11-GBq/μmol-specific activity. Sadly, however, this ligand shows low specific binding in combination with high nonspecific binding and is metabolized rapidly, rendering the tracer unsuitable for quantitative applications in human studies. This is a known disadvantage in brain studies but has a major impact on imaging applications in the vasculature, in which metabolites are an even greater complication.

### [^18^F]Nifene

[^18^F]Nifene [(S)-3-((2,5-dihydro-1H-pyrrol-2-yl)methoxy)-2-[^18^F]-fluoropyridine] was synthesized and evaluated for the first time in an animal model in 2006 by Pichika et al. [63]. Since then, the same group reported an optimized radiosynthesis (with 6–12 % in-hand yield, 10–20 % decay-corrected) and more preclinical data, particularly in rats and rhesus monkeys [64, 65]. So far, their research has shown [^18^F]nifene to have rapid brain kinetics and reasonable binding potential (1.7), whereas its binding in the brain (but not the cerebellum) could be displaced by nicotine, indicating specific binding to a nicotinergic receptor, presumably α4β2. Although some prior optimization must be done to fully characterize radiometabolites and their in vivo behavior, this tracer appears to be an available candidate for human studies in the near future.

### [^18^F]FA

2-[^18^F]FA and 6-[^18^F]FA (respectively, (S)-3-(azetidin-2-methyloxy)-2-[^18^F]fluoropyridine and (S)-3-(azetidin-2methyloxy)-6-[^18^F]fluoropyridine), also known as 2-[^18^F]-A-85380 and 6-[^18^F]-A-85380, can be synthesized with a high non-decay-corrected yield of about 50 % [66]. Both are very polar compounds with a resulting moderate BBB permeation and slow brain uptake. In combination with the slow washout rates, requiring scanning up to 6-h post injection, these tracers are not optimal for brain research. They still are being used, however, owing greatly to their relatively good specific nAChR binding, and several publications have appeared both preclinically and clinically, even in the past few years. In comparing 2-FA with 6-FA, there is a general preference for 2-FA because of its lower toxicity (15 vs. 1.8 μmol/kg).

Also available is a single-photon emission CT (SPECT) version of the A-85380 tracer: [^123^I]-5-IA-85380 ([^123^I]-5-IA; [^123^I]-5-iodo-3-[2(*S*)-azetidinylmethoxy]pyridine). This compound has properties necessary for in vivo imaging of nAChRs with SPECT, including high affinity (dissociation constant [Kd] = 11 pmol/L), rapid entry into the brain, low nonspecific binding, and minimal toxicity. However, it has the disadvantage of using an isotope suitable only for SPECT, with its intrinsic lower resolution and lack of good quantification possibilities, but like the ^18^F-labeled versions, also shows a slow washout rate [67].

The slow clearance of [^18^F]FA from the blood (half-life, 6 h) poses several challenges for vasculature imaging, as late scan times often are impractical (for patients) and less accurate (because of potential metabolite build-up). Similar ligands, such as [^18^F]nifzetidine and [^11^C]MA, showed similarly slow kinetics, rendering this class of ligands feasible but suboptimal for human use [63, 64, 68].

### [^18^F]ZW-104

[^18^F]ZW-104 [(S)-3-(azetidin-2-ylmethoxy)-5-(6-[^18^F]fluorohex-1-ynyl)pyridine] is a selective ligand for the β2-containing nAChRs and has a better binding affinity profile than 2-FA [69]. Radiosynthesis is performed in two steps, followed by HPLC purification, and shows rather poor yields (3–5 %) and moderate specific radioactivity. Brain uptake in baboons is high and specific (demonstrated by the blocking effect of nicotine) and is better overall than that of [^18^F]FA; however, it still is characterized by slow kinetics, especially during the washout.

### [^18^F]AZAN-α

[^18^F]AZAN was developed as an improvement over the 2-[^18^F]FA and 6-[^18^F]FA ligands, aiming to increase brain uptake and washout kinetics while maintaining a high binding affinity [70, 71]. The precursor currently is available commercially, and although the synthesis yield is similar to that of [^18^F]FA (around 50 %), the one-step synthesis is less complex than the two-step synthesis of [^18^F]FA compounds (although HPLC purification still is required), making the radiosynthesis more robust.

After initial trials in baboons confirming specificity and rapid kinetics (90-min scan time is sufficient), human studies were conducted [72, 73]. Wong et al. [72] showed that [^18^F]AZAN has a favorable dosimetry (0.014 mSv/MBq) and rapidly enters the human brain, with maximal regional brain uptake before 20 min post injection. The binding potential was around 2.5 (slightly better than that of nifene and FA), and specificity was confirmed by full blockage of uptake by the smoking cessation drug varenicline, a selective α4β2-nAChR partial agonist. In terms of stability, three radiometabolites were found, one of which entered the brain. It remains to be investigated to what extent these would interfere in the peripheral signal.

### [^18^F]Flubatine

(−)-[^18^F]Flubatine [(−)-[^18^ F]norchloro-fluoro-homoepibatidine] can be produced with a good yield (60 %) and high specific activities of >750 GBq/μmol [49, 50••]. The ligand binds with high affinity and selectivity to the α4β2 receptor subtype. The tracer is very stable in vivo (nearly 90 % at 90 min post injections) and shows only moderate plasma protein binding (15 %). Although never tried in vascular imaging, this compound seems feasible for this indication.

Another functional enantiomer, (+)-flubatine, similar in binding characteristics to (−)-flubatine, has a somewhat higher toxicity (1.5 vs. 6.2 μg/kg for the (−) enantiomer). This toxicity is a known problem in epibatine analogues and in the case of [^18^F]FPH, with an LD_50_ of 40 nmol/kg, even forced discontinuation of research [74, 75].

## Current Tracers for α7-nAChRs

Compared with α4β2-nAChRs, the α7 receptor is much more difficult to visualize in the human brain, mainly because of its lower expression of about 50 fmol/mg protein [40]. Similar values were demonstrated in vascular endothelial cells, with expression levels varying between 53 fmol/mg protein (untreated cells) and 385 fmol/mg protein (cells exposed to 50-μM nicotine). The steric and electronic requirements of the orthosteric agonist-binding site of the α7 receptor are fulfilled by structurally diverse classes of compounds. Currently, the most promising agents for molecular imaging in vivo are the substituted diazabicyclononane derivatives. One of these compounds, [^11^C]CHIBA-1001, has been investigated thoroughly in preclinical and preliminary clinical studies. Similar ^18^F-labeled compounds also have been quite successful, and the full inventory of currently developed functional radioligands is described in the following text.
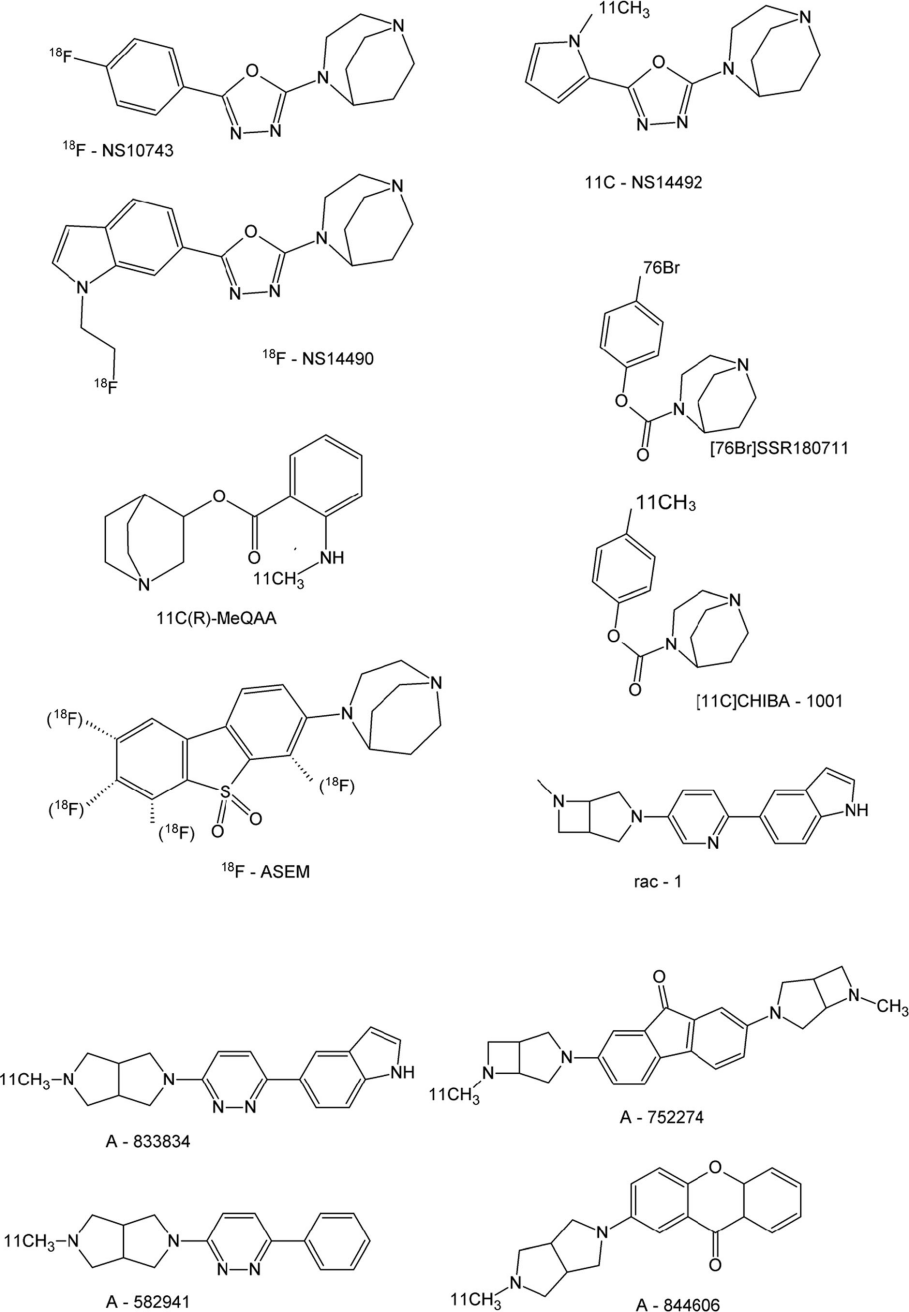


### [^11^C]A-752274

[^11^C]A-752274 and [^11^C]A-833834 both were developed by the group of Horti et al. [76]. The radioligands were prepared with a radiochemical yield of 12 to 32 %, high specific radioactivity (300–400 GBq/μmol), and radiochemical purity >95 %. Biodistribution studies in mice with [^11^C]A-833834 showed low specific α7-nAChR binding in the brain, and although [^11^C]A-752274 specifically (∼50 %) labeled α7-nAChR in mouse thalamus, it exhibited low uptake in baboon brain, thereby raising questions about its efficacy.

### [^11^C]A-582941 and [^11^C]A-844606

[^11^C]A-582941 and [^11^C]A-844606 were developed in 2009 by the group of Toyohara et al. [77]. The ligands showed specific uptake in brain regions with high α7 expression; however, they also were unstable, showing rapid formation of two metabolites, rendering these tracers unsuitable for peripheral imaging.

### [^11^C]rac-(1)

[^11^C]rac-(1) was developed by Gao et al. [78]. The radiosynthesis was accomplished via *N*-methylation of the nor-methyl precursor followed by semipreparative HPLC purification and ^18^C solid-phase extraction and formulation, resulting in an average yield of 30 %, radiochemical purity >98 %, and specific activity of 444 ± 74 GBq/μmol. Affinity toward the α7-nAChR is about 0.5 nM, which allows in vivo use. In the mouse brain, [^11^C]rac-(1) specifically accumulated in the frontal cortex and hippocampus, regions with an elevated density of α7-nAChRs, whereas uptake in the cerebellum, a region with low α7-nAChR densities, was nonspecific. Further research in monkeys, including in vivo displacement studies, are required before human research is performed, but so far the compound seems promising.

### [^11^C]MeQAA

Compared with some of the previously described ^11^C-labeled compounds, (R)-2-methylamino-benzoic acid 1-aza-bicyclo[2.2.2]oct-3-yl ester ([^11^C]MeQAA) is more stable and does not produce any metabolites that enter the brain [79]. This tracer shows an intermediate affinity for the α7-nAChR (41 nM) in vitro and a (rhesus monkey) brain distribution pattern in vivo that matches the α7 expression distribution, but so far no displacement studies have been performed.

### [^76^Br]SSR180771 and [^11^C]CHIBA1001

[^11^C]CHIBA1001 and its bromide analogue, [^76^Br]SSR180771, were developed in 2008 by the group of Hashimoto et al. [80–82], with further trials conducted in later years. [^76^Br]SSR180771 was prepared with an intermediate yield of 17 % and with high radiochemical purity and specific activity (respectively, 100 % and 8 GBq/μmol). For [^11^C]CHIBA1001, the yield was around 10 %. Although the affinity of these compounds for α7-nAChR is not optimal (120–180 nM), the uptake of both compounds in rhesus monkeys was fast and matched α7 distribution patterns. Furthermore, the brain uptake could be reduced significantly by using an α7-receptor specific agonist (SSR180771), but not by an α4β2-receptor specific ligand (A85380), indicating specific uptake. Because of the cost of ^76^Br and the higher brain uptake of the ^11^C analogue, preference is given to [^11^C]CHIBA for human research. So far, a limited human study with [^11^C]CHIBA showed high in vivo plasma stability (>80 % at 60 min post injection) and the highest brain distribution volume in the thalamus, although regional differences in brain activity were small [83]. The rather poor affinity of this ligand contraindicates further human research.

### [^18^F]NS10743

[^18^F]NS10743 was first reported in 2009 by Deuther-Conrad et al. [84, 85], who produced the tracer from a nitro precursor. The specific activity exceeded 150 GBq/μmol at a radiochemical purity >99 %. This tracer showed a relatively high affinity of about 10 nM toward α7 and about 84 nM toward α3β4 in vitro and partially reduced the cortical and hippocampal binding of tritiated α-bungarotoxin ex vivo. In vivo, [^18^F]NS10743 showed rapid uptake in mouse brain, which could be reduced by SSR180711, a known specific α7 ligand, thereby proving in vivo specificity.

### [^18^F]ASEM

As a response to poor-affinity ligands such as [^11^C]CHIBA, [^18^F]ASEM (3-(1,4-diazabicyclo[3.2.2]nonane-4-yl)-6-[^18^F]fluorodibenzo[b,d]thiophene-5,5-dioxide) was designed [86]. In total, the group of Gao et al. [86, 87] developed five ligands, each differing by position of the fluorine atom. Based on in vitro assays and in vivo PET and biodistribution studies, they concluded that [^18^F]ASEM (with the ^18^F in ortho position from the sulfoxide structure and away from the diazabicyclononane) showed the most promising characteristics for imaging. [^18^F]ASEM is produced with a typical yield of about 16 % (non-decay-corrected) and shows a specific activity of 300–1200 GBq/μmol and >99 % radiochemical purity. Care must be taken during the purification process: HPLC is required to remove the nitro precursors completely, as they also have a high affinity toward the α7 receptor. The radioligand itself is characterized by a subnanomolar affinity toward α7-nAChR (0.4 nM) and a very high selectivity compared with other nicotinergic receptors [86, 87]. In vivo, the brain distribution pattern in mice matched that of known α7-receptor expression and could be decreased significantly by SSR180771. So far, no human research has been performed with this ligand, although it appears promising for vascular research.

### [^18^F]NS14490 and [^11^C]NS14492

[^18^F]NS14490 and [^11^C]NS14492, developed by the group of Brust et al. [54••, 55, 88], can be synthesized with a 70 % yield and show a good affinity toward α7-nAChR (about 2.5 nM). Sadly, however, brain uptake is rather low in mice, casting doubt about their utility for brain research.

Although human data showed mediocre applicability of this ligand for brain α7-nAChR imaging, imaging of vascular α7-nAChRs has proven feasible [54••]. The mediocre α7 binding in the brain may be the result of poor BBB permeation or perhaps of efflux processes by P-glycoprotein, features less relevant when imaging in the periphery [54••, 55].

## Conclusions

During the past several years, interest has been growing in the use of nAChRs for diagnosing as well as treating neurologic, oncologic, and vascular disorders. Specific imaging of neuronal and non-neuronal nAChRs therefore is crucial, and PET imaging offers a noninvasive and easily applicable methodology. Although several nAChR PET tracers are available today, most are still in preclinical evaluation or in clinical evaluation for imaging of cerebral nAChRs in the context of neurodegenerative disorders. However, initial attempts have been made to apply these tracers to the imaging of non-neuronal α7- and α4β2-nAChRs in the cardiovascular system. The results of these feasibility studies, both in humans and in the experimental setting, show great promise, so further steps toward clinical implementation of in vivo vascular nAChR PET imaging in atherosclerosis should be taken. Therefore, research is under way to further evaluate the tracers available for clinical imaging of non-neuronal nAChRs, particularly in patients with known atherosclerosis in the carotid but also in the coronary arteries, and to develop new PET tracers for different nAChRs, with the goal of proving the feasibility of nAChR PET imaging in the clinical context.
